# High expression of signal regulatory protein beta 2 marks a favourable prognostic AML subgroup and associates with increased sensitivity to phagocytosis

**DOI:** 10.1007/s12026-025-09659-w

**Published:** 2025-06-25

**Authors:** Nienke Visser, Nisha K. van der Meer, Yuan He, Gerwin Huls, Jan Jacob Schuringa, Edwin Bremer

**Affiliations:** 1 Department of Hematology, University of Groningen, University Medical Center Groningen (UMCG), Groningen, The Netherlands; 2 Department of Basic Medicine, School of Basic Medicine and Clinical Pharmacy, Pharmaceutical University, Nanjing, China

**Keywords:** SIRP-ß2, AML, Phagocytosis, Innate immune targeting

## Abstract

Acute myeloid leukaemia is an aggressive hematologic malignancy that remains exceedingly difficult to treat despite recent advancements. High expression of CD47 on leukemic blasts interacts with the inhibitory receptor SIRP-alpha (SIRP-α) on innate immune cells, resulting in a strong "don't eat me" signal. Therefore, identifying AML patients who could benefit from immune-targeted therapies is crucial SIRP-β2 is predominantly expressed in myeloid cells and positively regulates innate anticancer immunity. Furthermore, endogenously expressed SIRP-β2 potentiates cancer cell trogocytosis by granulocytes. Here, we delineate the role of SIRP-β2 in AML. High expression of SIRP-β2 is independently associated with favorable overall survival (OS) and event free survival (EFS) independent of the ELN intermediate risk group. SIRP-β2 is more prevalent in the more committed FAB M4 and M5 subgroups. SIRP-β2 is also expressed on normal myeloid cells in patient samples, with higher expression on tumor-suppressive M1 macrophages than on adverse prognostic and tumor-supportive M2 macrophages. In line with this, co-culture of macrophages/neutrophils with ectopically expressed SIRP-β2 tumor cells results in an increased phagocytosis/trogocytosis treated with anti-CD47. These data indicate that AML patients with high SIRP-β2 AML expression could significantly benefit from innate immune-targeting therapies such as CD47 immune checkpoint inhibitor.

**Supplementary Information:**

The online version contains supplementary material available at 10.1007/s12026-025-09659-w.

## Introduction

Acute myeloid leukaemia (AML) is an aggressive myeloid malignancy with a median overall survival of 16.6 months. Immune evasive mechanisms such as the Signal Regulatory Protein alpha (SIRP-α)/CD47 axis are thought to play a pivotal role in the adverse survival [1. Unfortunately, therapeutic targeting of this pathway in AML using Magrolimab, a CD47 blocking antibody that inhibits this reaction, has yielded disappointing results in clinical trials, resulting in termination of some of the studies [2, 3]. In B cell lymphomas, targeting of CD47 was only efficient upon combination therapy with an ‘eat me’ signalling drug, such as the antibody Rituximab (RTX) [4, 5]. Thus, modulation of the phagocytic balance of AML should not only focus on ‘don’t eat me’ signals, such as SIRP-α/CD47, but also on delineating the impact of potential ‘eat me’ signals.

In this respect, we recently reported on a previously uninvestigated member of the SIRP family, SIRP-β2, which promoted innate antitumor immunity by macrophages and neutrophils in vitro [6].

## Results and discussion

**Fig. 1:**
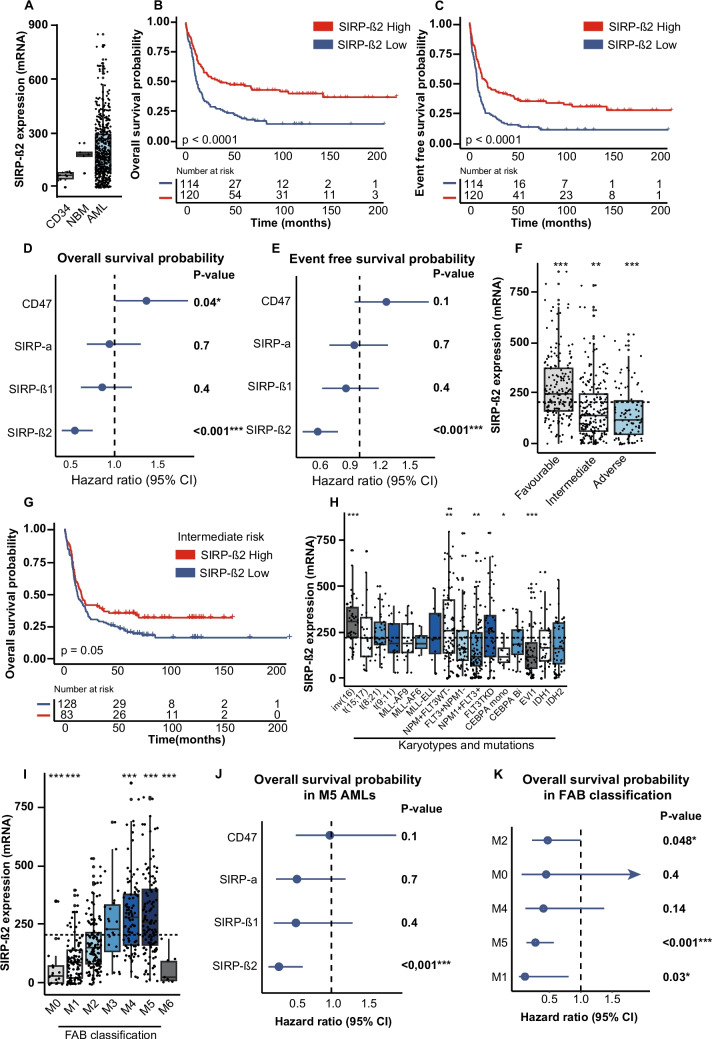
**Expression of CD47 and SIRP-α do not, but SIRP-β2 does correlate with patient’s overall survival.** **A.** SIRP-β2 expression on CD34, NBM and AML of the GSE6891 set (*n* = 234). **B.** Kaplan meier plot of the OS and EFS (**C**) of AML patients with the upper two (SIRP-β2^High^) and lower two (SIRP-β2^Low^) quartiles (*n* = 234). **D.** Multivariate cox regression of OS and EFS (**E**) on SIRP-β1, SIRP-β2, SIRP-α and CD47. **F.** SIRP-β2 expression in ELN risk groups: favourable, intermediate and adverse risk groups. **G.** Kaplan meier plot of the OS in the ELN intermediate risk group of AML patients with the upper two (SIRP-β2^High^) and lower two (SIRP-β2^Low^) quartiles. **H.** Up or downregulation of SIRP-β2 expression in various karyotypes and mutations of AML. **I.** SIRP-β2 expression divided amongst FAB classification (M0-M6). **J.** Multivariate of OS survival probability in M5 AMLs in CD47, SIRP-α and SIRP-β1. **K**. Univariate of OS probability in FAB classification. All boxplots compare the individual groups versus the other groups, t-test and Mann Whitney U test where used for parametric and non-parametric statistical analysis. p values are indicated as: ***p < 0.001, **p < 0.01, and *p < 0.05

On AML cells, the endogenous expression of SIRP-β2 could be an ‘eat me’ signal relevant to the phagocytic balance governing innate immune responses. Indeed, SIRP-β2 expression was detected at the mRNA level in 234 de novo AML patients from the GSE6891 set, with MDS and APL patients being excluded (Fig. 1A). With an variable expression profile amongst AML patients when compared to healthy CD34 + blasts and normal bone marrow (NBM) (Fig. 1A).

When analysing AML patients in the upper (SIRP-β2^High^) expression quartile, these patients had a favourable overall survival (OS) (HR: 0.61; CI; 0.492–0.771; *p*- < 0.0001) and event free survival (EFS) (HR: 0.668; CI: 0.539–0.827; *p*- < 0.001)(Fig. 1B, C) compared to patients in the lower expression quartile (SIRP-β2^Low^). SIRP-β2 expression retained its significant impact on favourable outcome in Cox proportional hazards regression analyses and positively predicted for OS (Fig. 1D) and EFS (Fig. 1E) in both uni- and multivariate analyses, independent of age, European Leukemia Net (ELN)–risk stratification 2017 and hematopoietic stem cell transplantation (HSCT) (Suppl. Table 1). In the TCGA AML dataset, derived from BloodSpot (n = 172), high expression of SIRP-β2 also associated with a favourable survival (Suppl. Figure 1A, *p* = 0.019) [7], thereby, validating SIRP-β2 as independent predictor in different cohorts. In contrast, although SIRP-β1 is also reported as a co-stimulatory member of the SIRP family [8], its expression level did not associate with prognosis (Fig. 1D, E, Table 1). Similarly, SIRP-α expression did not associate with prognosis, although CD47 did have a significant negative predictive value (Fig. 1D, E, Suppl. Table 1), consistent with literature [9]. Thus, within the SIRP family, only SIRP-β2 significantly and independently associated with prognosis in AML.

According to the ELN2017 risk classification of the GSE6891 set, SIRP-β2 expression was highest in the favourable risk group and lowest in the adverse risk group (Fig. 1F). Differentially expressed SIRP-β2 specifically predicted a favourable OS of patients classified in ELN2017 intermediate risk group (HR 0.73; CI: 0.525–1.00; *p* = 0.05) (Fig. 1G), whereas SIRP-β2 expression did not associate with survival in patients in the adverse and favourable risk group (Suppl. Figure 1B, C). When classifying AML based on molecular aberrations, expression of SIRP-β2 was significantly higher in the inv(16) and *NPM1*cyt/*FLT3*-*ITD*wt subgroup. Interestingly, SIRP-β2 expression was significantly lower in *NPM1cyt* patients with a concomitant FLT3-ITD mutation (Fig. 1H), which suggests SIRP-β2 expression levels may be affected by a FLT3-ITD induced block in differentiation [10]. When classifying based on the FAB classification, SIRP-β2 expression was significantly higher in the more committed FAB M4 and M5 subgroups than in M1 to M3 subgroups (Fig. 1I). Within the more committed FAB M5 subgroup, SIRP-β2^high^ was a powerful determinant of favourable prognosis in contrast to CD47, SIRP-α and SIRP-β1 (Fig. 1J, Suppl. Figure 1D). As well as in the low expressing M1 and M2 subgroups (Fig. 1K) suggesting that SIRP-β2^high^ expression also predicts a favourable outcome independent of the maturation state of the blasts. Altogether, high SIRP-β2 expression, identifies a more committed type of AML, that is either sensitive to current treatment strategies, or prone to innate antitumor immunity induced by the SIRP-β2 ‘eat me’ signal. Thus, this may identify a set of patients that would conceivably be sensitive to innate immune checkpoint inhibitors (ICI), where the SIRP-β2 ‘eat me’ signal has an additive anti-tumour response with the ‘don’t eat me’ blockade. In line with this, CD47-targeting monoclonal antibody Magrolimab did not improve clinical response in the adverse risk (FAB: M1) TP53 mutant immature AMLs [2], a group in which based on our findings the majority is SIRP-β2^low^ and lacks an additive ‘eat me’ signal. Thus, stratifying patients by SIRP-β2 high expression could guide specific treatment choice, e.g. FAB M1, SIRP-β2^high^ patients possibly benefit from anti-CD47 treatment. Reversely, SIRP-β2^low^ patients might benefit from standard treatments, such as monotherapy with hypomethylating agents or combination therapy with Venetoclax (VEN), with VEN being less effective in committed AML blasts and more effective towards immature blasts [11].

**Fig. 2:**
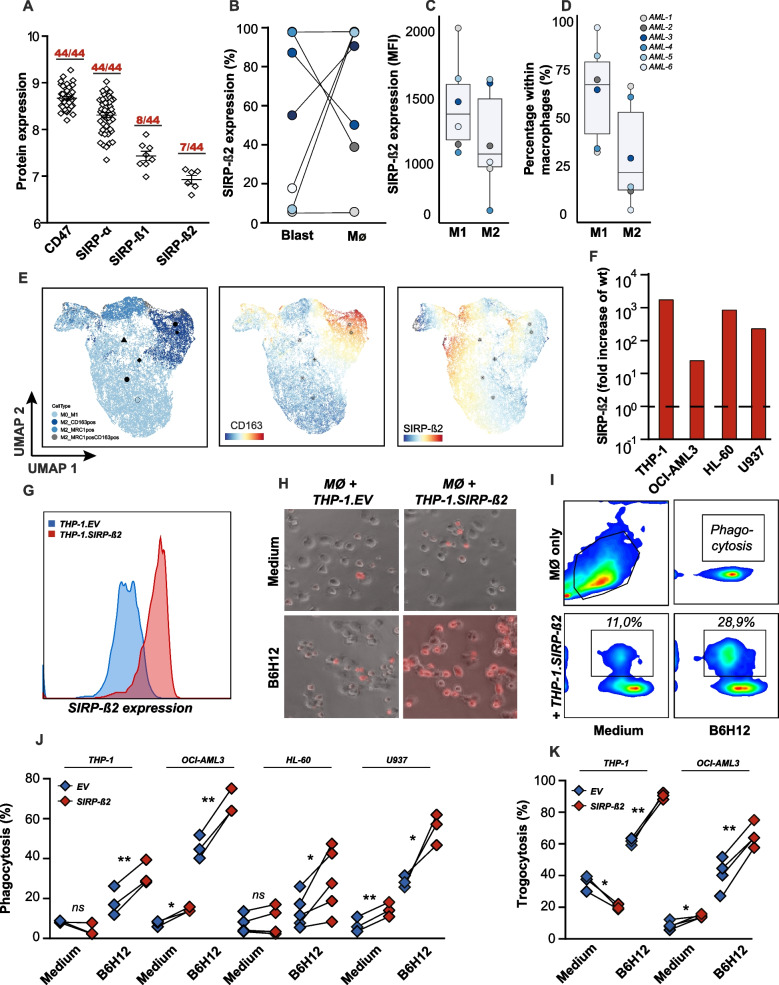
**Ectopic expression of SIRP-β2 in hematopoietic cancer cell lines augments their macrophage-mediated phagocytic removal upon anti-CD47 antibody treatment.** **A**. Protein expression of CD47, SIRP-α, SIRP-β1 and SIRP-β2 as determined by mass spectrometry analysis of membrane extracts of 44 primary AML samples. **B**. SIRP-β2 expression on blasts cells and macrophages derived from AML patients (n = 8), determined by flow cytometry. **C**. SIRP-β2 expression on M1 and M2 macrophages of 6 AML samples, determined by flow cytometry **D**. Percentages of M1 and M2 on the total macrophage compartment of 6 AML samples, determined by flow cytometry. **E**. Uniform manifold approximation projection (UMAP) based on flow cytometry data, projecting the different types of macrophages of 6 AML samples. Two UMAPs projecting the florescent intensity of SIRPB2 and CD163 of the macrophages. **F**. Quantification of SIRP-β2 in overexpressed AML cell lines (U937, THP-1, OCI-AML3 and HL-60), using RT-qPCR. **G**. Ectopic expression of SIRP-β2 of THP-1, determined by flow cytometry. **H**. Illustrative images of phagocytosis of THP-1.EV and THP-1.SIRP-β2 cells after 3 h co-cultured with monocyte-derived macrophages, including medium control and co-treatment with B6H12. **I**. Gating strategy of monocyte-derived macrophages in combination with THP-1.SIRP-β2, using flow cytometry. **J**. Quantification of monocyte-derived macrophage phagocytosis of THP-1.EV/THP-1.SIRP-β2, OCI-AML3.EV/OCI-AML3.SIRP-β2, HL-60.EV/HL-60.SIRP-β2 and U-937.EV/U-937.SIRP-β2. **K**. Quantification of granulocyte trogocytosis of THP-1.EV/THP-1.SIRP-β2 and OCI-AML3.EV/OCI-AML3.SIRP-β2. Student’s t-test: p values are indicated as: **p < 0.01, and *p < 0.05

Using proteomics, endogenous expression of SIRP-β2 at protein level was detected in 7 out of 44 primary AML samples (LFQ2015 dataset within CD34 + compartment)(Fig. 2A). Interestingly, expression of SIRP-β2 was detected (in vitro*)* at the cell surface of blasts as well as macrophages of AML patients, varying between 0–100% of the cells (Fig. 2B, Suppl. 1E). This expression pattern is in line with mRNA expression reported above (Fig. 1A). For a more in depth understanding of the particular cell types expressing SIRP-β2, spectral flow cytometry using 21 markers was performed on 6 AML samples. SIRP-β2 expression was more pronounced on tumor suppressive M1 macrophages than on the adverse prognostic tumor supportive M2 macrophages (Fig. 2C). Those patients with higher percentages of M2 macrophage had lower SIRP-β2 expression (AML 3, AML 5) on the surface of M2 macrophages (Fig. 2D). This pattern was also evident on the 2D Uniform manifold approximation projection (UMAP) visualization of the macrophage landscape, in which SIRP-β2 levels were downregulated on CD163^high^ tumour-supportive M2 polarized macrophages (Fig. 2E). Notably, in our previous report we already demonstrated that healthy SIRP-β2^high^ expressing macrophages have higher phagocytic activity. Although not directly investigated here, these data would suggest that, in the setting of AML, CD163^low^/SIRP-β2^high^ macrophages could associate with favourable prognosis, whereas reversely CD163^high^/SIRP-β2^low^ macrophages could associate with poor prognosis. Unlike the macrophages there was no clear distinction of SIRP-β2 expression among the different phenotypes of blasts (Suppl. Figure 1 F, G). Thus, in addition to SIRP-β2 expression on leukemic cells, the surrounding macrophages in the tumor microenvironment (TME) may benefit from immune-supportive effects of SIRP-β2.

To further comprehend the favourable impact of elevated SIRP-β2 in the context of innate checkpoint inhibition, macrophage-mediated phagocytosis was evaluated using ectopically expressing SIRP-β2 AML cell lines, as validated both for mRNA and surface expression (Fig. 2F, G). Upon mixed culture of THP-1.SIRP-β2 and control THP-1.EV with monocyte-derived macrophages, a slight yet non-significant increase in basal level of cancer cell uptake by macrophages was detected (Fig. 2H, I, 11,0%). However, upon treatment with CD47 antibody B6H12 phagocytosis of THP-1.SIRP-β2 by macrophages was increased by almost 30% compared to THP-1.EV (Fig. 2H, I, B6H12, 28,9%). In line with this data, significantly higher phagocytic uptake of SIRP-β2 expressing cell lines upon treatment with B6H12 compared to EV controls was detected in all AML cell lines (*p* < 0.01 for THP-1 and OCI-AML3; *p* < 0.05 for HL-60 and U-937) (Fig. 2J). Further, a significant increase in baseline phagocytosis was detected in 2 out of 4 of the AML cell lines ectopically expressing SIRP-β2 (Fig. 2J). These findings provide evidence for our previous speculations that the presence of more ‘eat me’ signal through SIRP-β2 on cancer cells can potentiate therapeutic inhibition of the CD47/SIRPa “don’t eat me” signal on macrophages. To further explore the impact of AML-expressed SIRP-β2 on immunity, co-cultures were performed with polymorphonuclear cells (PMNs), the most prevalent immune cell in the blood that is comprised for ~ 95% of neutrophils [12, 13]. A main effector function of granulocytes is trogocytosis, with basal trogocytosis of ~ 20% with THP-1^SIRP−β2^ and ~ 10% of OCI-AML3^SIRP−β2^, respectively, compared to 40% and 10% trogocytosis with THP-1^EV^ and OCI-AML3^EV^ control by isolated granulocytes (*p* < 0.05)(Fig. 2K). However, trogocytosis of ectopic SIRP-β2-expressing cell lines was significantly enhanced by 20–30% compared to EV controls upon treatment with CD47 antibody B6H12 (*p* < 0.001)(Fig. 2K). Notably, SIRP-β2 itself does not bind to CD47 (Suppl. Figure 1H) and thus likely does not directly impact B6H12 interaction, with the binding partner of SIRP-β2 as yet unidentified.

## Conclusion

Taken together, SIRP-β2 is broadly expressed in AML patients and high expression of SIRP-β2 independently associates with favourable OS and EFS independent of the ELN intermediate risk group and FAB classification. A survival benefit that is presumably induced by higher susceptibility to phagocytosis observed in SIRP-β2^high^ AML blasts. Conversely high SIRP-β2 expression on non M2-macrophages enhances phagocytosis of tumor cells [6], suggesting a potential SIRP-β2-SIRP-β2 interaction or an increased cytokine production by SIRP-β2 macrophages, that drives an ‘eat me’ signal in SIRP-β2^high^ patients. Alternatively, both pathways might be mutually exclusive, where AML patients either have SIRP-β2^high^ blasts or SIRP-β2^high^ macrophages. However, regardless of the exact mechanism underlying the additional phagocytosis and trogocytosis upon treatment with CD47 antibody B6H12 specifically in SIRP-β2^high^ AMLs, these data suggest that SIRP-β2^high^ AML patients could significantly benefit from innate immune targeting therapies such as CD47 immune checkpoint inhibitor (ICI).

## Methods

### Cell lines and patient derived AML cells

Cell lines THP-1, OCI-AML3, HL-60 and U937 were obtained from the American Type Culture Collection (ATCC, Manassas, VA) and cultured according the manufacturing conditions. All cell lines were cultured at 37 °C in a humidified 5% CO_2_ containing atmosphere.

Patient-derived AML samples (stored after informed consent and following approval by the Medical Ethical Committee of the UMCG in accordance with the Declaration of Helsinki protocol code NL43844.042.13, 6 January 2014) were thawed from cryovials and added to pre-warmed newborn calf serum (NCS, Gibco, Breda, The Netherlands), and centrifuged at 450* g* for 5 min. Thereafter, the pellet was re-suspended in pre-warmed NCS mix (4 µM magnesium sulphate (Sigma-Aldrich, St. Louis, MO), 20U/ml DNase (Roche, Basel, Switzerland) and 5U/ml Heparin (Pharmacy of the UMCG, The Netherlands) and incubated at 37 °C for 15 min and harvested (5 min, 450 g). AML cells were washed and cultured in Gartner’s medium (Alpha-MEM, 12.5% horse serum, 12.5% FCS, 1 µM hydrocortisone (Sigma-Aldrich, St. Louis, MO, USA), 1% pen-strep (Sigma-Aldrich, St. Louis, MO, USA), and 50 µM beta-mercaptoethanol (Sigma-Aldrich, St. Louis, MO, USA)) supplemented with thrombopoietin (TPO) and G-CSF, IL-3 (20 ng/mL of each cytokine) (hospital pharmacy, UMCG).

### Protein expression of SIRP-β2 on AML samples

SIRP-β2 expression was determined on AML samples, following. 1.0 × 10^5^ cells were taken from the AML sample and were incubated with SIRP-β2 antibody (#P61637, Thermo Fisher Scientific, Waltham, MA, USA) for 1 h incubation at 4 °C washed 3 times with PBS, followed by incubation with Goat-anti-Rabbit-488 (#A-11034, Thermo Fisher Scientific, Waltham, MA, USA) for 30 min at 4 °C. Cell were washed 3 times with PBS, the expression was examined using Cytoflex (Beckman Coulter, Brea, CA, USA).

RNA expression was measured as described in our previous paper, [6]*.*

### Macrophage phagocytosis/granulocyte trogocytosis

Healthy peripheral blood mononuclear cells (PBMCs) were used to isolate granulocytes and monocytes using ficoll density gradient (Lymphoprep™, Bernburg, Germany). Monocytes were isolated from the mononuclear fraction, using CD14 microbeads following the manufactures protocol (Miltenyi Biotec, Leiden, The Netherlands). The isolated monocytes were first differentiated into macrophages and then further polarized into M1-like macrophages, using 20 ng/mL IFNy and 50 ng/mL LPS (ImmunoTools, Friesoythe, Germany) for 24 h.

5.0 × 10^4^ macrophages were co-cultured with Incucyte Cytolight Red labelled tumor cells, according to manufacturer’s instructions at an effector to target (E:T) ratio of 1:5 and treated with anti-CD47 (1 µg/mL) for 3 h at 37 °C. The macrophage phagocytosis was examined using Cytoflex (Beckman Coulter, Brea, CA, USA).

For granulocyte trogocytosis: The pellet after lymphoprep was further used for granulocyte isolation, by resuspending the pellet in 1X red blood cell lysis (Sigma Aldrich, St. Louis, MO, USA) for 15 min at RT and washed twice with PBS. Granulocytes were also co-cultured with the Incucyte Cytolight Red labelled tumor cells at an E:T ratio of 1:1 and treated with anti-CD47 (1 µg/mL) for 3 h at 37 °C. The granulocyte trogocytosis was examined using Cytoflex (Beckman Coulter, Brea, CA, USA).

### Statistical analysis

Statistical significance was determined using a Student’s t-test and Mann Whitney U test was used for parametric and non-parametric statistical analysis. p values are indicated as: *** *p* < 0.001, ** *p* < 0.01, and * *p* < 0.05.

## Supplementary Information

Below is the link to the electronic supplementary material.

Supplementary file1 (PDF 969 KB)

Supplementary file2 (PDF 397 KB)

## Abbreviations

AML: Acute myeloid leukaemia
ELN: European Leukemia Net
EFS: Event free survival
HSCT: Hematopoietic stem cell transplantation
ICI: Immune checkpoint inhibitor
NBM: Normal bone marrow
OS: Overall survival
RTX: Rituximab
SIRP-α: Signal Regulatory Protein alpha
TME: Tumor microenvironment
UMAP: Uniform manifold approximation projection
VEN: Venetoclax
