# Supplementary figures and images for: High expression of signal regulatory protein beta 2 marks a favourable prognostic AML subgroup and associates with increased sensitivity to phagocytosis
Source: Immunol Res. 2025 Jun 25;73(1):98. doi: 10.1007/s12026-025-09659-w (PMC12198062; doi:10.1007/s12026-025-09659-w)

Suppl. Figure 1

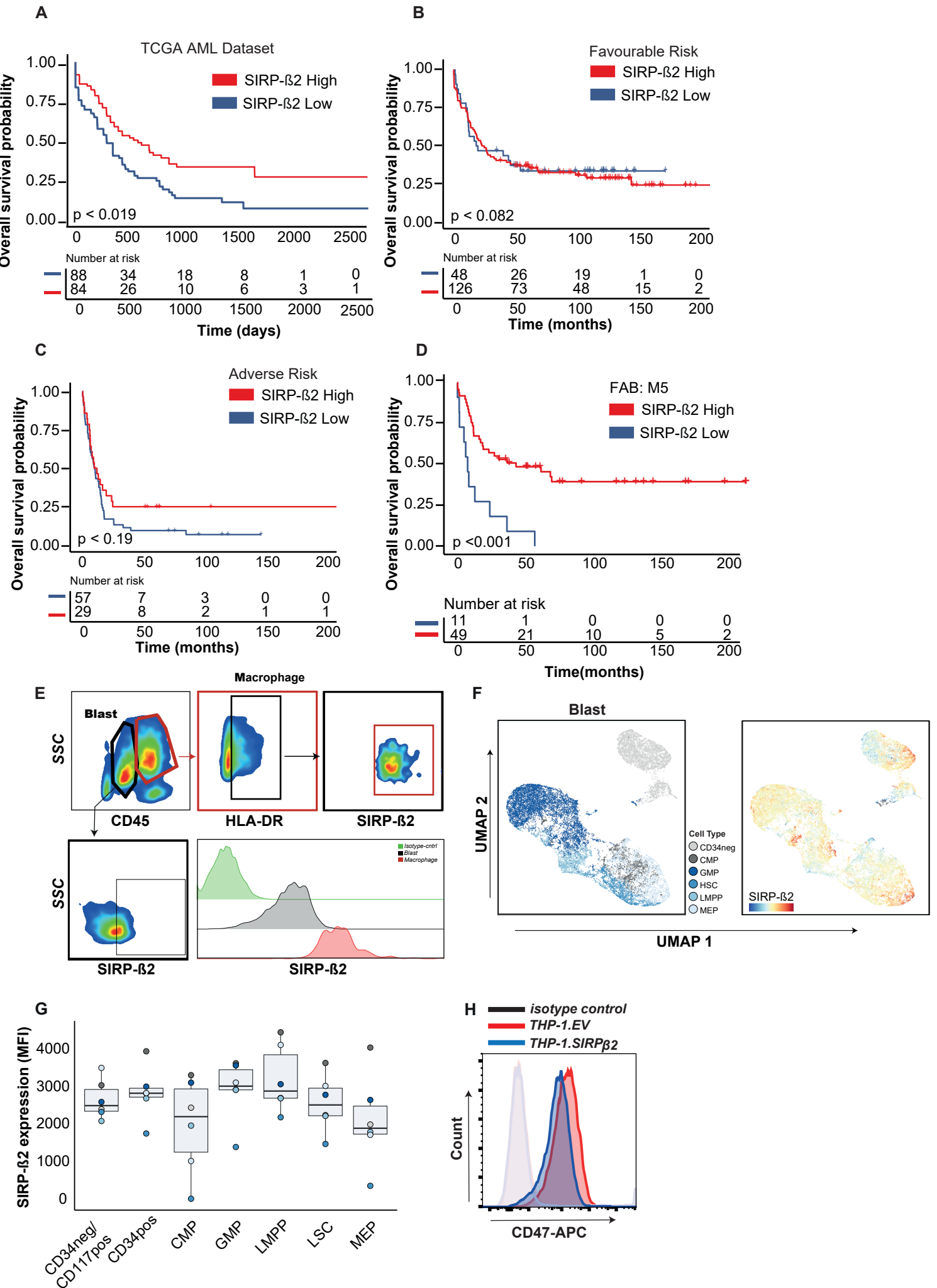

Supplement: Supplementary file 1 — Supplementary file1 (PDF 969 KB) [file 12026_2025_9659_MOESM1_ESM.pdf]
