# Supplementary material for: High expression of signal regulatory protein beta 2 marks a favourable prognostic AML subgroup and associates with increased sensitivity to phagocytosis
Source: Immunol Res. 2025 Jun 25;73(1):98. doi: 10.1007/s12026-025-09659-w (PMC12198062; doi:10.1007/s12026-025-09659-w)
Supplement: Supplementary file 2 — Supplementary file2 (PDF 397 KB) [file 12026_2025_9659_MOESM2_ESM.pdf]

Suppl. Table 1

Univariate and multivariate analysis - AML patients

	Univariate			*Multivariate*		
	**Hazard ratio**	**95%CI**	**P-Value**	**Hazard ratio**	**95%CI**	**P-Value**
**Overall survival**						
**Age (continuous)**	1.017	1.008 - 1.025	<0.0001****			
**HSCT (categorized)**	0.5291	0.420 - 0.665	<0.0001****			
Autologous or allogeneic						
No						
**ELN2017 (categorized)**	1.903	1.457 - 2.484	<0.0001****			
Adverse risk						
Intermediate risk						
Favorable risk						
**SIRP-β2**	0.616	0.492 - 0.771	<0.0001****	0.691	0.550 - 0.868	**0.001***
**SIRP-β1**	0.913	0.730 - 1.140	0.421	0.941	0.751 - 1.177	0.593
**SIRP-α**	0.825	0.661 - 1.030	0.089	0.821	0.658 - 1.026	0.083
**CD47**	1.227	0.982 - 1.533	0.072	1.332	1.065 - 1.667	**0.012***
**Event free survival**						
**Age (continuous)**	1.010	1.002 - 1.018	0.01**			
**HSCT (categorized)**	0.663	0.534 - 0.823	0.001***			
Autologous or allogeneic						
No						
**ELN2017 (categorized)**	2.061	1.593 - 2.667	<0.0001****			
Adverse risk						
Intermediate risk						
Favorable risk						
**SIRP-β2**	0.668	0.539 - 0.827	**<0.001***	0.721	0.581 - 0.894	**0.003**
**SIRP-β1**	0.902	0.729 - 1.116	0.344	0.907	0.732 - 1.124	0.372
**SIRP-α**	0.874	0.707 - 1.081	0.215	0.867	0.701 - 1.072	0.188
**CD47**	1.229	0.994 - 1.521	0.057	1.330	1.073 - 1.648	**0.009**
